# Transition from Spin Dewetting to continuous film in spin coating of Liquid Crystal 5CB

**DOI:** 10.1038/s41598-018-25504-7

**Published:** 2018-05-08

**Authors:** Palash Dhara, Nandini Bhandaru, Anuja Das, Rabibrata Mukherjee

**Affiliations:** Instability and Soft Patterning Laboratory, Department of Chemical Engineering, Indian Institute of Technology Kharagpur, Kharagpur, Pin-721302 India

## Abstract

Spin dewetting refers to spontaneous rupture of the dispensed solution layer during spin coating, resulting in isolated but periodic, regular sized domains of the solute and is pre-dominant when the solute concentration (***C***_***n***_) is very low. In this article we report how the morphology of liquid crystal (LC) 5CB thin films coated on flat and patterned PMMA substrate transform from spin dewetted droplets to continuous films with increase in ***C***_***n***_. We further show that within the spin dewetted regime, with gradual increase in the solute concentration, periodicity of the isotropic droplets (***λ***_***D***_) as well as their mean diameter (***d***_***D***_), gradually decreases, till the film becomes continuous at a critical concentration (***C***_n_*). Interestingly, the trend that ***λ***_***D***_ reduces with increase in ***C***_***n***_ is exact opposite to what is observed in thermal/solvent vapor induced dewetting of a thin film. The spin dewetted droplets exhibit transient Radial texture, in contrast to Schlieren texture observed in elongated threads and continuous films of 5CB, which remains in the Nematic phase at room temperature. Finally we show that by casting the film on a grating patterned substrate it becomes possible to align the spin dewetted droplets along the contours substrate patterns.

## Introduction

Thin films coated on solid surfaces find wide applications as adhesives^1^, biological membranes^2^, organic photovoltaics^3^, modulation of optical properties of surfaces and so on^4^. However, long term stability of such films, particularly the ones thinner than 100 nm on a non wettable surface is a non-trivial scientific issue, and has been subject to extensive research for more than two decades^5–10^. Disjoining pressure arising out of interfacial van der Waals’ interaction within the film leads to the growth of thermally excited surface capillary waves with time, leading to spontaneous rupture and dewetting of ultra-thin films, particularly on non wettable surfaces^6^. While such instability is undesirable from the standpoint of coatings^11^, pattern formation associated with it is gaining popularity as a viable non lithographic alternative for creating meso and nano structures, particularly on a topographically or chemically patterned substrate, which results in perfect ordering of the features^5,12–17^.

While initial experiments on spontaneous instability of ultra-thin films involved simple homo-polymers such as PS, PMMA^5–17^, dewetting of complex materials such as liquid crystal (LC) has also received significant attention. Dewetting of a LC thin films is intriguing due to the coupling of the structural effects arising from the anisotropic and oriented nature of the molecules along with the destabilizing van der Waal’s forces active in films thinner than ≈100 nm^18–34^. Anisotropic anchoring of the LC molecules at the film-substrate interface (weak planar anchoring) and the free surface (strongly homeotropic anchoring) gives rise to additional elastic force field within the thin film^24^. Additionally, a thermotropic LC thin film undergoes phase transformation with temperature, due to variation in the directional and positional order of the LC molecules^18^. Combination of all these factors result in a far more complex morphological evolution scenario in thin LC films, particularly during thermal annealing^23,25–29^. Apart from the free surface system, the molecular alignment and ordering within a confined liquid crystal system is widely studied by theoretically^35–40^ and experimentally^41–44^. The alignment of liquid crystal molecules strongly depends on the boundary conditions whether the LC film is confined between two plates or with a free surface on a substrates. Uniform alignment of LC mesogens is achieved on a modified soft substrates. The modification of soft polymeric substrates was done by creating nano or micro grooves using rubbing process^45–47^, scanning by AFM tip^48^, making microstructures by lithography^49–52^. The boundary plates of a confined system not only play a role for local anchoring or molecular ordering at the interfaces but for entire thin layer. The complex ordering and phase behavior of LC layer within a confined system strongly depends on the interplay between short range surface interaction and long range intermolecular forces^53,54^. The local anchoring or ordering and stability is strongly depends on absorption and wetting behavior of LC mesogens on the substrates^55^. A details understanding of the relationship between orientation alignment of LC molecules and the substrates interface is very helpful not only for LC device application but for fundamental studies of bulk LC properties. Several papers have described the ordering of 5CB molecules by wetting or absorbing on different substrates^56–58^. LC thin films are also important due to their role in various applications such as vapor^59^, and biological sensors^60^, actuators for artificial muscles and so on^61^, in addition to display technology^62^, and therefore understanding their stability is a matter of great scientific importance.

In this article, we focus on liquid crystal 5CB (4-n-pentyl-4′-cyanobiphenyl), which is a thermotropic LC that remains in the Nematic phase (N) at the room temperature. Thin films of 5CB have been subject to several thermal dewetting studies. When heated, undulations appear on the surface of a 5CB thin film as it undergoes Nematic to Isotropic (I) phase transformation close to the N to I phase transformation temperature (***T***_***N***−***I***_) or the clearing point^26–28^. In fact, the N–I phase transformation occurs over a temperature range, which depends on the thickness of the film^28^. Within this temperature window both N and I phases co-exist at equilibrium during the forward (N → I) as well as the backward (I → N) transformations encountered during gradual heating or cooling of the film^28^. Incidentally, these undulations were initially confused as signature of spinodal dewetting in several early studies^22–25^. Herminghaus *et al*. subsequently showed that spontaneous rupture and true dewetting of a 5CB film occurs only deep within the Isotropic phase, when the 5CB film is heated close to about 70 °C, a temperature that is way above the ***T***_***N***−***I***_ (≈33 °C)^29^. However, unlike a homopolymer thin film, where the dewetted structures remain permanent even when the film is cooled below the glass transition temperature (***T***_***g***_) of the polymer, the dewetted LC droplets rewet the surface and heal the ruptured film during cooling^29^. Recently, phase transformation as well as reversible dewetting of a 5CB film has been observed by subjecting the film to solvent vapor by Bandyopadhyay and co-workers^30^. While we focus on 5CB thin films, for the sake of completeness it is worth highlighting that LC thin films that remain in the Smectic phase at room temperature have additional stabilizing effect due to ordering of Smectic layers oriented parallel to the substrate^31–34^. Vix *et al*. showed cross over between dewetting and stabilization depending on whether at least three smectic layers have laterally organized or not^34^.

Apart from thermal annealing^5–10^, solvent vapor exposure^63^, or quenching the film in a mix of solvent and non-solvent^64^, which are the commonly adopted approaches for engendering instability in a thin polymer film, a novel approach is to achieve dewetting during spin coating itself^65–69^. The method relies on the rupture of an extremely dilute solution layer on the substrate during spin coating itself, forming isolated solute patches. While spin dewetting is totally undesired from the stand point of achieving a continuous thin film, it is finding potential utilization as a rapid meso fabrication technique for solution processable materials. Spin dewetting has already been utilized for fabricating array of polymers (PS and PMMA) droplets on topographically patterned substrate^65–69^. Very recently by Bandyopadhyay *et al*. utilized spin dewetting to obtain an ordered array of LC droplets on a physico-chemically patterned substrate^70^.

Interestingly, most of the published papers on spin dewetting report rupture and dewetting of a solution layer over a topographically patterned substrate, where preferential rupture takes place along the contours of the substrate patterns. Till date there has been no a single study on spin dewetting over a smooth, homogeneous substrate which shows complete disintegration of the film down to droplets. In this article, we show for the first time the formation of isolated 5CB droplets on flat and patterned PMMA substrates due to spin dewetting of a dilute 5CB solution. The spin dewetted morphology comprises of an isotropic array of nearly equal sized droplets with a dominant periodicity (***λ***_***D***_), and is morphologically very similar to a droplet array obtained from dewetting of thin films. We report how ***λ***_***D***_, mean droplet diameter (***d***_***D***_) and droplet number density (***N***_***D***_) vary with increase in ***C***_***n***_ within the spin dewetted regime. We also show that at a particular RPM, the film morphology gradually transforms from array of isolated droplets, to threads and eventually a continuous film with gradual increase in ***C***_n_^*^. We finally show that the spin dewetted droplets can be aligned on a topographically patterned substrate with grating geometry where significant reduction in both ***λ***_***D***_ and ***d***_***D***_ is achieved as a consequence of the lateral confinement imposed by the substrate patterns.

## Results and Discussions

**Figure 1:**
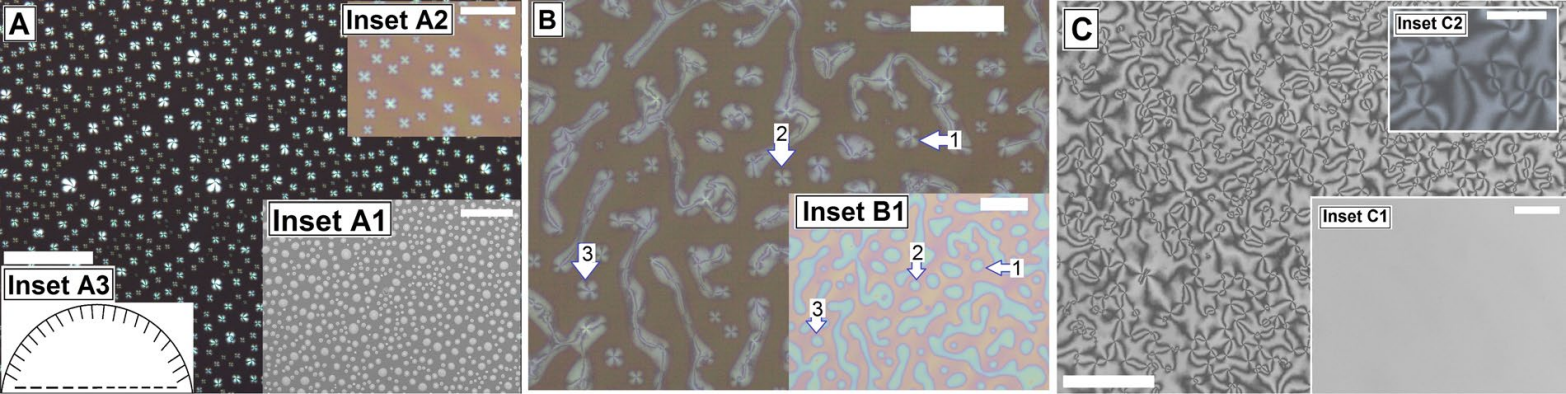
Cross polarized microscope images of (**A**) spin dewetted 5CB droplets obtained for **C**_**n**_ = 1.0%; (**B**) Transition morphology comprising of both droplets and threads for **C**_**n**_ = 1.25%; and (**C**) Continnuous film for **C**_**n**_ = 3.0%. Insets A1, B1 and C1 shows the corrsponding bright field images. Inset A2 and C2 show higher magnification images under cross polarized light. Scale bar 100 µm in all images. Inset A3 shows indicative radial anchoring of the 5CB molecules along the periphery of an hemispherical spin dewetted droplet.

Figure 1 shows the gradual transition in morphology of the as cast film with increase in ***C***_***n***_. The morphology changes from an array of isolated spin dewetted droplets for ***C***_***n***_ upto 1.0%, a transition morphology comprising collection of droplets and elongated threads for ***C***_***n***_ = 1.25% and a continuous film for ***C***_***n***_ ≥ 1.5%. From Fig. 1A we calculate ***λ***_***D***_ ≈ 11.55 ± 1.19 μm and ***d***_***D***_ ≈ 5.84 ± 1.04 μm for the spin dewetted droplets. The continuous film in Fig. 1C (h ≈ 132 ± 2.7 nm) exhibits well known Schlieren texture, with 4 brushes (black lines) originating out of every point singularity. From inset C2, Fig. 1C where a magnified view of the texture is presented, it can be clearly observed that the morphology is similar to Nematic Schlieren texture with inversion lines of the first kind^71^. The appearance of such texture is attributed to planar degenerate alignment of the 5CB molecules on the PMMA substrate, which tries to impose uniform orientation on the 5CB molecules. On the other hand, as the nature of anchoring at the free surface of the film is homeotropic (***θ***_***e***_ = 0), there is a continuous change in the director field within the film from the base to the free surface, which results in the Schlieren texture^71^. Our observation with films of different thickness reveals that the length scale of the texture increases with enhanced film thickness (Figure S1, Supporting Information). As the film becomes thinner, the director field changes more rapidly along the depth of the film to match the antagonistic anchoring between the two boundaries, resulting in higher number of brushes per unit area of the film^71^.

**Figure 2:**
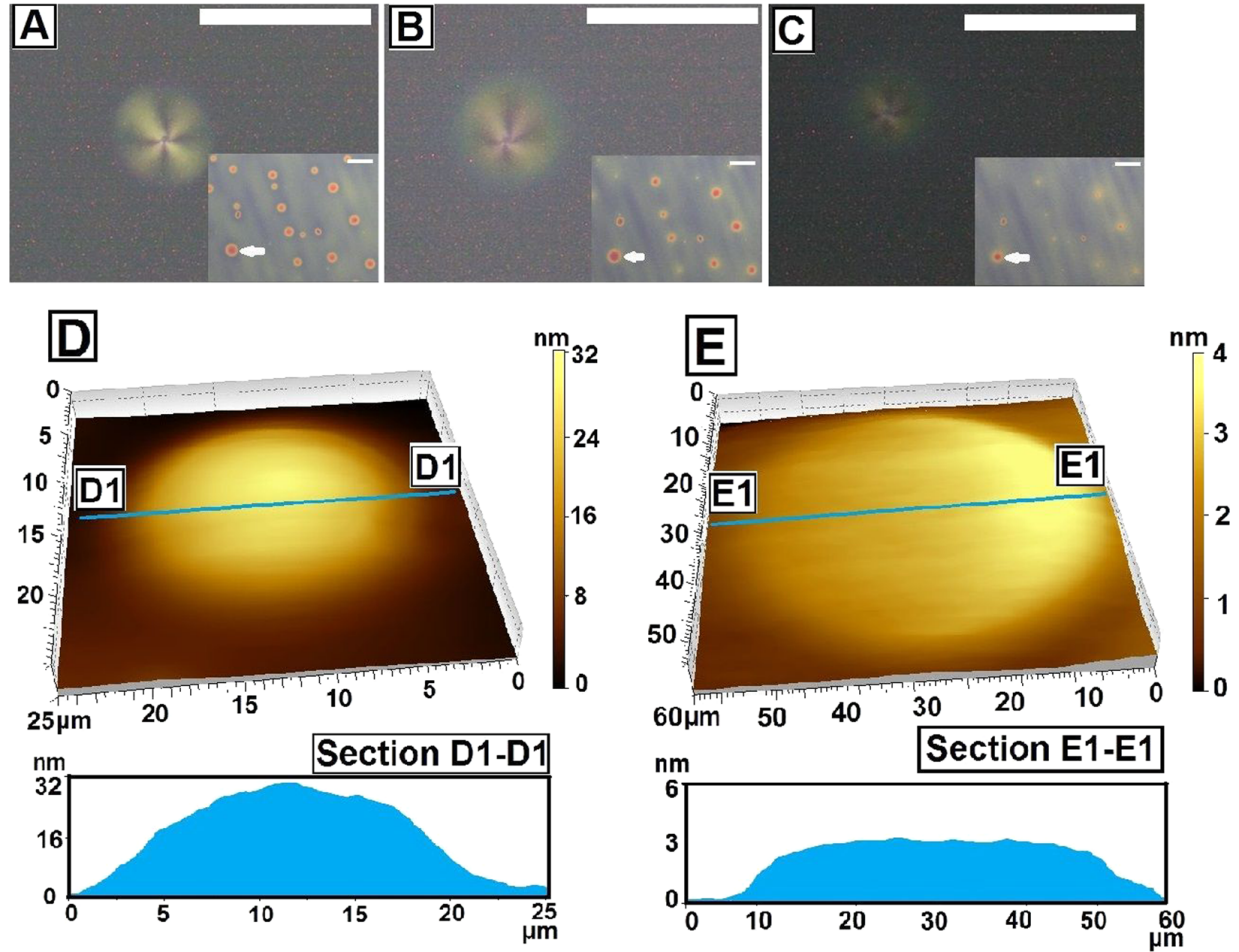
Gradual disappearance of Radial texture in a spin dewetted 5CB droplets with time. A single droplet is shown under crossed polarized microscope after (**A**) 1 min, (**B**) 52 min (**C**) 75 min. The insets show bright field images of the same sample over a large area, with the specific droplet shown in the main frame is marked with an arrow. The scale bar is 50 µm in all frames. (**D**,**E**) AFM images of the droplet after2 min and 80 min respectively.

Next we focus our attention on Fig. 1A, which shows that the spin dewetted droplets exhibit Radial texture. This is attributed to hometropic anchoring along a hemispherical free surface (inset A3, Fig. 1A)^59,60,72^. We observe the appearance of Radial textures in all spin dewetted droplets obtained at different ***C***_***n***_, including in the droplets seen in Fig. 1B. Radial texture in 5CB drops has earlier been observed in drop cast 5CB films on solid surfaces^59^, as well as in 5CB droplets dispensed on liquid^60^. Interestingly, Radial textures in the droplets gradually disappear with time, which is discussed in details in the context of Fig. 2. Control experiments reveal that spin dewetted droplets formed on other surfaces such as silicon wafer and cross linked PDMS (Figure S2, supporting information), also exhibit Radial texture though the number density of the droplets as well as the shape of the texture varies on different substrates. A detailed study of variation of texture of the spin dewetted droplets on different substrates is beyond the scope of the present article and will be taken up separately. While 5CB exhibits planar and planar degenerate anchoring on silicon wafer and PMMA substrates respectively, it exhibits homeotropic anchoring on crosslinked PDMS^73^. Thus, it becomes clear that the appearance of the Radial texture in a hemispherical 5CB droplets is independent of the nature of anchoring of the LC molecules on the substrate and is governed by the shape of the feature. This claim is further corroborated in Fig. 1B, which shows the transition morphology. In the figure it can be clearly seen that the texture varies as a function of feature geometry. While the threads in Fig. 1B exhibit Schileren texture, Radial texture is exhibited by the droplets (marked with arrows 1, 2 and 3). It is well known that conic structures, particularly focal conic domains (FCD) forms in LC films that are in Smectic A phase, where the molecules have both directional and positional order^74^. The formation of FCD is attributed to anisotropic anchoring condition at the two boundaries of the LC layer, along with positional ordering of the molecules^75^. A continuous Nematic 5CB film coated on a PMMA substrate however fails to exhibit conic domains due to lack positional order of the molecules. In contrast, the appearance of conic structure in the form of Radial texture in spin dewetted 5CB droplets imply positional ordering of the molecules within the hemispherical droplets, arguably imposed by the geometry of the droplet itself. We feel that the ***θ*** symmetric geometry of a hemispherical droplet imposes radial symmetry on the gradual transition in the director filed from the substrate to the free surface of the droplet, and consequently only the molecules at the apex of the droplets are perpendicular to the substrate. This results in the formation of a single pole, engendering Radial texture within the droplets. In contrast in a thread, the ***θ*** symmetry is broken along with loss of positional order of the molecules, which in turn results in Schileren texture.

We have already mentioned that the Radial texture of the spin dewetted droplets gradually disappear with time. This is shown in the first three frames (A–C) of Fig. 2, where a droplet is seen to shrink in size and nearly disappear after ~75 minutes. To gain deeper insight, we performed AFM imaging of the droplets shown in frames D and E of Fig. 2. From these images it can be clearly seen that the droplet spreads with time, which is also associated with reduction of height of the droplet. Spreading of a 5CB droplet on a polar (PMMA) substrate is expected, as the end group of 5CB (-CN) gets absorbed on the polar surface^20^. As previously reported by Valignat *et al*., the surface adsorbed lone 5CB molecule gets covered by an homeotropic inter digitated bilayer of 5CB molecules, due to the formation of quadrupole with 5CB molecules with heads facing each other^20^. This precursor film gradually spreads on the surface, which has orientation close to the surface normal. As the height of the drop reduces, the gradual transition in the direction of the director from the free surface to the substrate no longer takes place and consequently the Radial texture is lost. As the droplets spread and become very thin, they become almost invisible under an optical microscope (Inset C1, Fig. 2C), but could still be imaged with an AFM (though the images become increasingly noisy and often difficult to capture). AFM image in Fig. 2E shows the thickness of the spreading precursor layer to be ≈3.8 ± 0.3 nm, which is in good agreement with the thickness of 5CB pre-cursor layer reported previously^20^. In this regard we must highlight a precaution that must be taken to avoid experimental artifact. In typical experiments involving time dependent morphological evolution, generally the light source of the optical microscope is kept turned on during the entire duration of the experiment. However, for an LC droplet or a thin film this is enough for localized heating of the sample, which we found was enough to engender phase change in 5CB that has ***T***_***N***−***I***_ ≈ 33 °C. To circumvent this problem, the light source of the optical microscope was turned on only while taking the images, keeping it switched off at all other times. Following the latter protocol, we found that the Radial textures disappeared after ≈75 minutes, in contrast to its disappearance just after ≈14 minutes when the light was kept on. We also observed that the time necessary for disappearance of the Radial texture remains the same irrespective of whether the drop was kept open to atmosphere or covered under an inverted petri dish, indicating evaporation had no role on the disappearance of Radial texture.

**Figure 3:**
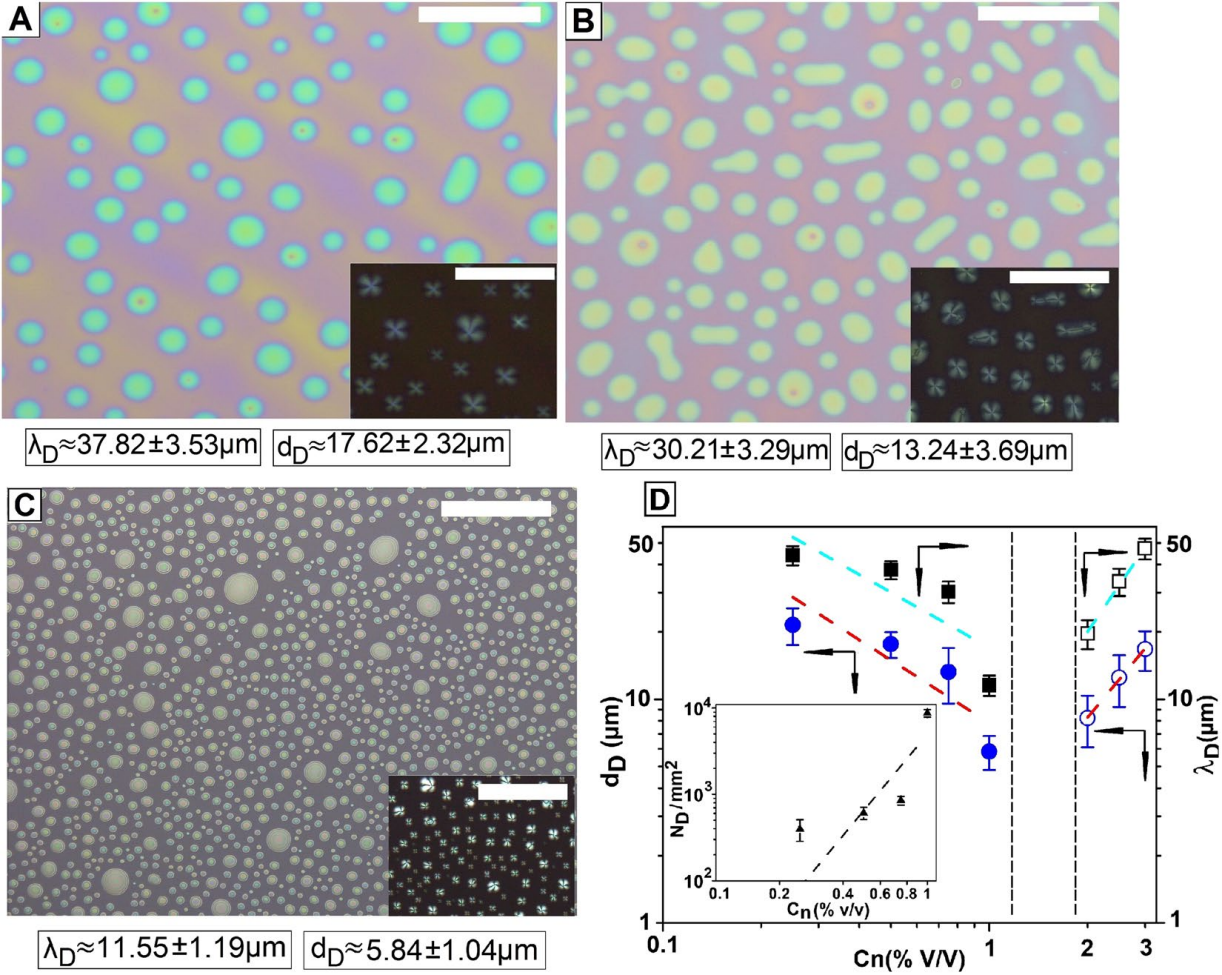
Spin dewetted droplets (**A**) **C**_**n**_ ≈ 0.5% (**B**) **C**_**n**_ ≈ 0.75% and (**C**) **C**_**n**_ ≈ 1%. (Scale bar 100 µm) (**D**) Variation of **d**_**D**_ (Circle) and **λ**_**D**_ (Square) with **C**_**n**_. Inset shows the variation of **N**_**D**_ with **C**_**n**_. The closed and open symbols are for droplets resulting from spin dewetting and thermal dewetting respectively.

Figure 3A–C shows the variation in the morphology of the spin dewetted droplets with increase in ***C***_***n***_ from 0.5% to 1.0%. In order to clearly understand the subsequent discussion, we briefly discuss the mechanism of spin dewetted droplet formation. It is well known that after dispensing the solution and initiation of rotation during spin coating, the solution layer continues to gradually dry because of continuous evaporation of solvent^76^. This in turn leads to continuous increase in the intrinsic solute concentration (***C***_***ni***_) within the solution layer. In regular spin coating ***C***_***ni***_ attains the saturation concentration (***C***_***ns***_) before the solution layer becomes too thin. As ***C***_***ni***_ ≈ ***C***_***nS***_ and solvent further continues to evaporate, solute molecules start to phase separate from the solution phase and get deposited on the substrate, leading to film formation. In this regard, it has been shown by Sanat Kumar and co-workers that when a rapidly evaporating solvent is used (such as Ethanol, vapor pressure = 0.0595 bar), Marangoni instabilities are set up on the film surface due to quick evaporation of the solvent and associated temperature gradient formation^77^. On the other hand, with progressive evaporation of solvent, the viscosity of the solution layer also increases and consequently, the Marangoni instability patterns get frozen on the surface of the film^77^. The situation becomes more complex in spin dewetting, that is when the solute concentration ***C***_***n***_ is very low. In such a situation, ***C***_***ni***_ fails to attain ***C***_***nS***_ even in the late stage of the spinning process. In case the thickness of the solution layer becomes thinner than ≈100 nm, the undulations arising out of Marangoni Instability grow due to disjoining pressure, resulting in rupture of the film during spinning. Subsequent retraction of the three phase contact line results in isolated patches of the solution layer over the substrate, within which all subsequent deposition of the solute takes place resulting in spin dewetted droplets.

The ***λ***_***D***_ and ***d***_***D***_ of the droplets is mentioned in the respective frames, for each case. The variation of ***λ***_***D***_ and ***d***_***D***_ as a function of ***C***_***n***_ is shown in Fig. 3D, for a constant RPM (=2200). The variation in ***N***_***D***_ with ***C***_***n***_ is shown as inset of Fig. 3D. We note that more number of smaller droplets appears as ***C***_***n***_ increases and consequently both ***λ***_***D***_ as well as ***d***_***D***_ of the spin dewetted gradually reduces with increase in ***C***_***n***_. From the best fit to the data in Fig. 3D, we obtain the dependences as ***λ***_***D***_ ≈ ***C***_***n***_^−***0***.***82*±*0***.***29***^ and ***d***_***D***_ ≈ ***C***_***n***_^**−*0***.***93*±*0***.***32***^. The trend that both ***λ***_***D***_ and ***d***_***D***_ decrease with increase in ***C***_***n***_ is exactly opposite to what is observed in dewetting of a continuous thin film, where both ***λ***_***D***_ and ***d***_***D***_ increase with increase in ***h***, that is increase in ***C***_***n***_^5^. This can also be seen in Fig. 3D, where the points with open symbols are data from control experiments which are related to thermal dewetting of continuous 5CB films heated above ***T***_***I–N***_. In this regime, the dependence of ***λ***_***D***_ and ***d***_***D***_ with ***C***_***n***_ are obtained as ***λ***_***D***_ ≈ ***C***_***n***_^***2***.***14***±***0***.***14***^ and ***d***_***D***_ ≈ ***C***_***n***_^***1***.***75***±***0***.***06***^ from the best fit to the data. However, as it is customary in classical dewetting literature to present the dependency of ***λ***_***D***_ and ***d***_***D***_ as a function of ***h***, we re-evaluate the scaling relations in terms of ***h***, which turns out to be ***λ***_***D***_ ≈ ***h***^***3***.***13***±***0***.***67***^ and ***d***_***D***_ ≈ ***h***^***2***.***56***±***0***.***54***^. Linear stability analysis of spinodal dewetting as well as many experimental studies suggests a dependence of ***λ***_***D***_ ≈ ***h***^***2***^ for polymeric thin films^5–10,16^. A higher value of the exponent (>2) in the relation between ***λ***_***D***_ and ***h*** suggests the existence of additional stabilizing force, probably due to the structural effects of the LC molecules (a detailed discussion on this point is beyond the scope of the present manuscript). For the sake of completeness we also highlight that the thermally dewetted droplets do not exhibit Radial texture (as the LC is in isotropic phase) and the film heals upon cooling as has been reported earlier^29^.

To justify the gradual reduction of ***λ***_***D***_ with increase in ***C***_***n***_, we look into the expression of the dominant wavelength of Marangoni instability, which is given by ***λ*** = π***h*** $\sqrt{\frac{32}{{M}_{a}}}$, where ***h*** is the film thickness and ***M***_***a***_ is the Marangoni Number^78^. It is also known that ***M***_***a***_ increases with ***C***_***n***_. Thus it becomes obvious that ***λ*** reduces with increase in ***C***_***n***_, which further corroborates that spin dewetting is dominated by the amplification of Marangoni instability. This is in clear contrast to spinodal dewetting of a thin polymer film which results from amplification of thermally excited surface capillary waves, where ***λ*** is seen to scale as ***h***^***2***^ ^6^. Further, it can be found from the literature that Marangoni Instability in a thin film dominates when ***M***_***a***_ > 80. Assuming ***M***_***a***_ ≈ 80, and using the values of ***λ***_***D***_ shown in Fig. 3D, we could find out an approximate thickness when Marangoni Instability sets in during spin coating, which is seen to vary between ≈6 µm to 22 µm. Of course this calculation is extremely gross and does not include the effect of the variation of ***M***_***a***_ with **C*n***. However, even this gross calculation does indicate that the Marangoni Instability during spin dewetting sets in when the thickness of the solution layer is of the order of ≈10 µm during spinning.

**Figure 4:**
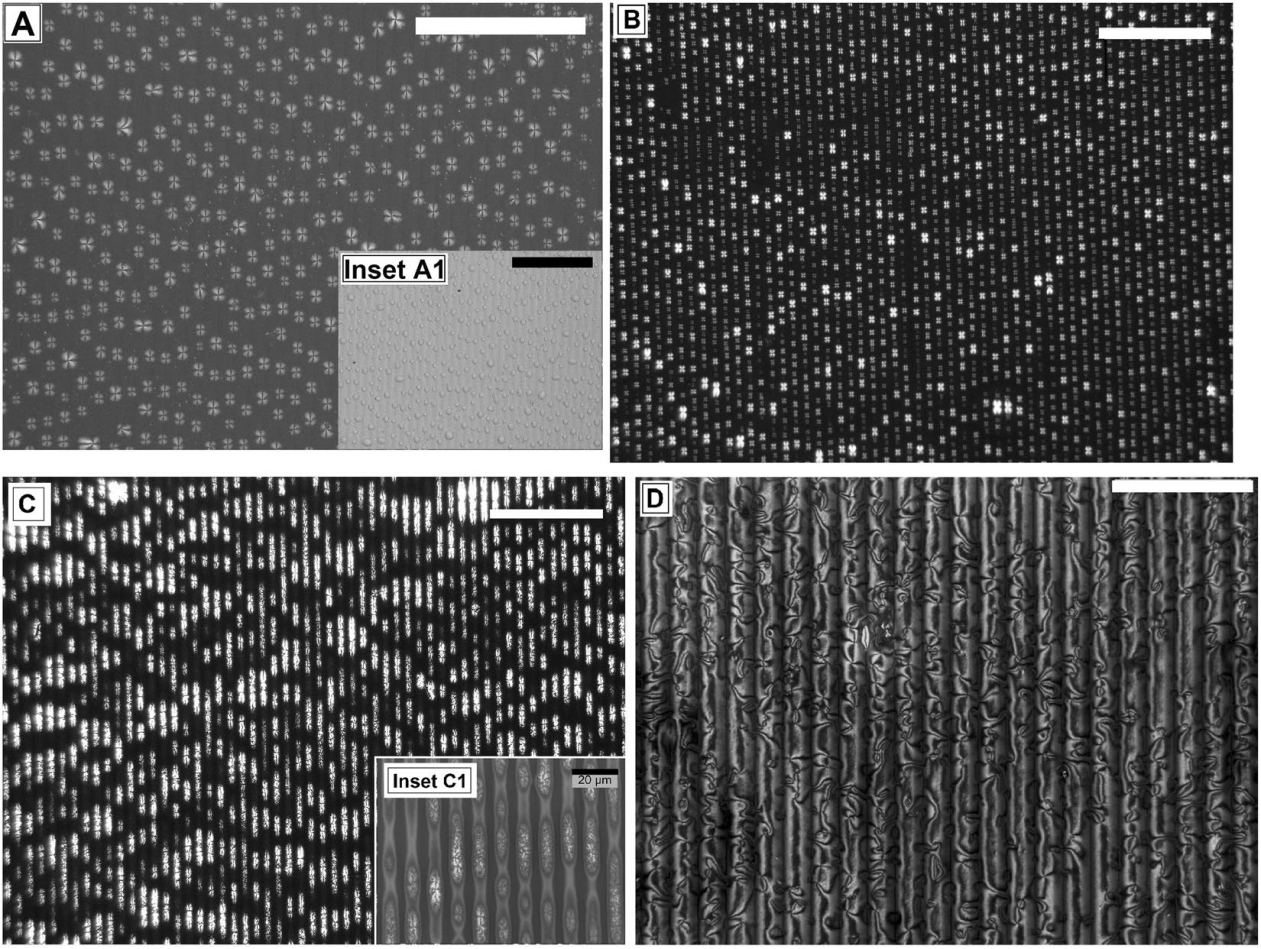
Morphology of 5CB spin coated on a patterned PMMA substrate. (**A**,**B**) Array of aligned spin dewetted droplets for **C**_**n**_ ≈ 0.5% and 1.75% respectively; (**C**) Aligned threads of 5CB for **C**_**n**_ ≈ 2.5% and (**D**) Continuous 5CB film for **C**_**n**_ ≈ 3.0%. Scale bar is 100 µm for frames (**A**–**C**) and inset A1; 50 µm for frame D.

In Fig. 4 we show the progressive variation in the as cast morphology of 5CB spin coated on a soft lithographically patterned PMMA substrate having grating geometry (line periodicity ***l***_***P***_ ≈ 20 µm, line height ***h***_***p***_ ≈ 150 nm, line width ***l***_***D***_ ≈ 10 µm). On a topographically patterned substrate, the thickness of the solution layer is thinnest over each substrate protrusion and consequently the film ruptures there. This result in isolated threads of solution layer confined within each groove. Depending on ***C***_***n***_, solute (5CB) may start to deposit covering the entire span of the groove (Fig. 4C) or the threads might further disintegrate due to a Rayleigh like instability into isolated droplets of the solution, eventually resulting into an array of periodically arranged spin dewetted droplets along the grooves (Fig. 4A and B)^66^. Apart from alignment and ordering, we observe significant downsizing of both ***λ***_***D***_ and ***d***_***D***_ on the patterned substrate. For example, for ***C***_***n***_ ≈ 0.50%, the ***λ***_***D***_ of the droplets reduces from ≈37.82 ± 3.53 µm on a flat substrate to ≈17.37 ± 1.54 µm on the patterned substrate, with associated reduction in ***d***_***D***_ is from ≈17.62 ± 2.32 µm to ≈6.56 ± 1.51 µm. The downsizing of ***λ***_***D***_ and ***d***_***D***_ on a patterned substrate clearly shows the effect of confinement on the size and periodicity of the spin dewetted features. Interestingly, the Radial texture in the spin dewetted droplets within the grooves last much longer (≈ 24 hours) before disappearance, as compared to that on a flat substrate. This is probably due to restricted spreading of the droplets in the transverse direction due to the presence of the substrate walls, which forces the drops to slowly spread only along the groove. Further, the trend that ***λ***_***D***_ of the droplets reduces with increase in ***C***_***n***_, as has been observed on flat substrates is also observed on patterned substrates. For example, ***λ***_***D***_ reduces from ≈17.37 ± 1.54 µm for ***C***_***n***_ ≈ 0.50% in Fig. 4A to 7.14 ± 1.38 µm for ***C***_***n***_ ≈ 1.75% in Fig. 4B.

With further increase of ***C***_***n***_ the morphology changes to threads aligned along each substrate groove, as can be seen in Fig. 4C for ***C***_***n***_ ≈ 2.50%. Interestingly the main frame of Fig. 4C under cross polarized light gives an apparent impression that the structures are discontinuous. However, from the higher magnification image of the same sample under polarized light (inset C1, Fig. 4C), it can be clearly seen that the apparently “isolated” patches are signature of transition in the texture due to coalescence of the drops into treads. Figure 4D shows a continuous film over the patterned surface obtained for ***C***_***n***_ ≈ 3.0%. While the film exhibits Nematic Schlieren texture, it is interesting to note that the brushes, which appear over each substrate feature, are also strongly oriented along the direction of the substrate patterns. This is probably attributed to the periodic variation in the thickness of a continuous film over a topographically patterned substrate^67^.

## Conclusion

To conclude, in this article we have looked into the fundamental aspect of spin coating a dilute solution of liquid crystal 5CB in ethanol on a PMMA substrate. Though PMMA is fully wetted by both the LC and the solvent, we show that a continuous film forms only above a critical solute concentration (***C***_n_*), at a constant RPM. For ***C***_***n***_ < ***C***_n_*, the solute fails to cover the entire substrate, resulting in an isotropic array of nearly equal sized hemispherical droplets due to spin dewetting. It is argued that Disjoining pressure induced late stage amplification of the Marangoni Instabilities on the film surface arising out of rapid evaporation of the solvent during spin coating is responsible for spin dewetting. The spin dewetted LC droplets exhibit Radial texture, indicating the presence of conic domain within them, in contrast to Schlieren texture observed in continuous 5CB films at room temperature. The Radial texture of the droplets disappear after ≈75 minutes, due to progressive spreading of the droplets on a PMMA substrate, where 5CB molecules form quadrupole with heads facing each other. We further show that the spin dewetted droplets can be aligned by spin coating on a topographically patterned substrate. Apart from ordering, significant miniaturization in both ***d***_***D***_ and ***λ***_***D***_ is achieved on the patterned substrate, in addition to much longer stability of the Radial texture within the droplets. With increase in ***C***_***n***_ the morphology progressively changes from closely packed array of droplets (lower ***λ***_***D***_), threads of LC covering every groove and finally a continuous film when ***C***_***n***_ ≥ ***C***_n_*. The value of ***C***_n_* on a patterned substrate is found to be much higher than that of a flat substrate, due to preferential rupture of the solution layer over the substrate protrusions. Even in a continuous film over a patterned substrates, the Schlieren texture is seen to be influenced by underlying substrate pattern.

## Methods

Dilute solution of 4-n-pentyl-4′-cyanobiphenyl (5CB, 99.99% pure Sigma Aldrich, Germany) with different levels of dilution (***C***_***n***_ = 0.5%, 0.75%, 1%, 1.25%, 1.5%, 2%, 2.5% and 3.0% (v/v)) in ethanol (Merck, Germany) was spin cast on flat and patterned PMMA substrates. The drop volume, rpm during spinning and spin durations were kept constant at 30 μl, 2200 rpm and 60 sec respectively in all experiments. The choice of substrate is extremely important for an LC thin film, as the anchoring on the substrate determines the texture of the film as well as influences its stability. As the 5CB molecules are strongly bipolar, a substrate with strong polarity is essential for obtaining a uniform LC thin film. On a non-polar substrate, an LC film will spontaneously dewet due to cohesive interaction among the polar molecules^22^. We found that PMMA is a preferred material for obtaining an LC thin film as the polar pendant ester groups present in PMMA helps in anchoring of the LC molecules. PMMA coated walls have been extensively used in LC cells, and it is well known that 5CB molecules exhibit “planar degenerate anchoring” on a PMMA substrate^79^. A substrate that allows 5CB to spread on it was essential, as we wanted to ensure that spin dewetting is happening due to the failure by the solute present to cover the entire substrate, and not due to cohesive interaction within the LC molecules. Consequently we used 2 μm thick films of PMMA dip coated on silicon wafer as the substrate. Ethanol was chosen as the solvent, as it does not dissolve or damage the PMMA substrate layer, which was verified from control experiments. In certain experiments, the PMMA layer was patterned by a pressure assisted Capillary Force Lithography, using a patterned cross linked polydymethylsiloxane (PDMS) stamp^80^.

The as-cast morphology of the 5CB droplets/films was characterized by optical microscopy (Leica DM 2500 M) in both bright field and cross polarized modes, as well as by Atomic Force Microscopy (AFM, Agilent Technology, USA, Model 5100). The thickness of the continuous films on flat substrates was measured using an imaging ellipsometer (Accuran GmbH, Model EP3). Optical micrographs were processed with open source image processing software, ImageJ to determine the droplet diameter (***d***_***D***_), number density of the droplets (***N***_***D***_) and periodicity of the droplets (***λ***_***D***_).

## Electronic supplementary material

Supporting Information
