# Supplementary material for: Transition from Spin Dewetting to continuous film in spin coating of Liquid Crystal 5CB
Source: Sci Rep. 2018 May 8;8:7169. doi: 10.1038/s41598-018-25504-7 (PMC5940909; doi:10.1038/s41598-018-25504-7)
Supplement: Supplementary file 1 — Supporting Information [file 41598_2018_25504_MOESM1_ESM.pdf]

Transition from Spin Dewetting to continuous film in spin coating of Liquid Crystal 5CB

Palash Dhara, Nandini Bhandaru, Anuja Das and Rabibrata Mukherjee*

Instability and Soft Patterning Laboratory, Department of Chemical Engineering, Indian Institute of Technology Kharagpur, Pin-721302, India.

*Author for correspondence. e-mail: rabibrata@che.iitkgp.ernet.in , Tel: +91-3222 283912

Online Supporting Information

S1.0 Variation in the Nature of Schlieren Texture in continuous films with gradual increase in Film Thickness

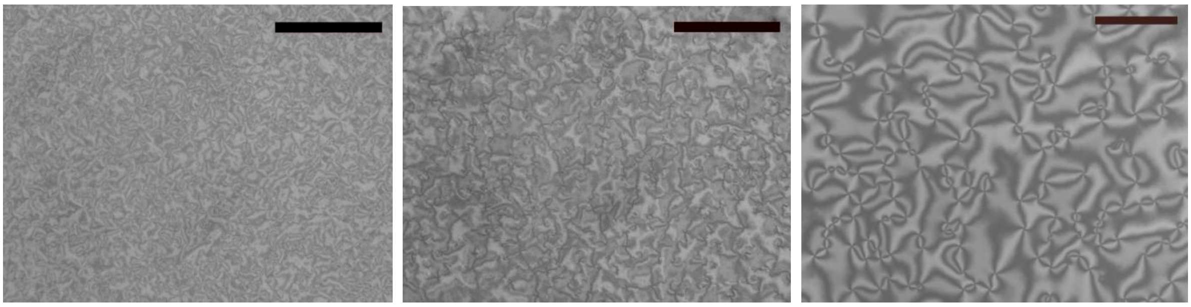

Figure S1: *Variation in the Nature of Schlieren Texture in continuous 5CB films with (a) thinness (h) = 69.5 ±1.8 nm; (b) h =92.8 ±4.3 nm and (c) h = 153.7 ±2.3 nm.*

S2.0 Spin Dewetted droplets on Various Substrates

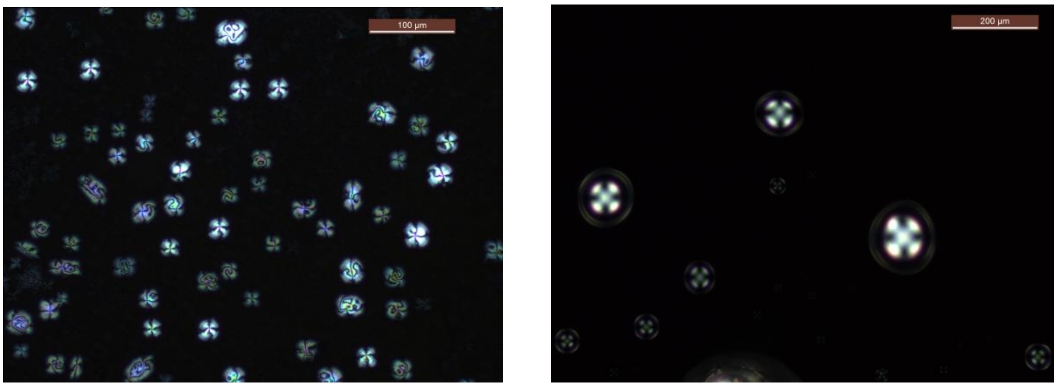

Figure S2: *Spin dewetted 5CB droplets with Radial Texture on (A) Silicon Wafer and (B) cross linked PDMS substrates.*
